# Effect of Mid-Adolescent Dietary Practices on Eating Behaviors and Attitudes in Adulthood

**DOI:** 10.3390/nu15010225

**Published:** 2023-01-01

**Authors:** Miao Wu, Lin Wu, Akira Ishida

**Affiliations:** Graduate School of Agricultural Science, Kobe University, Kobe 657-8501, Japan

**Keywords:** dietary practices, eating behaviors, eating attitudes, adolescence, childhood, adulthood, Japan

## Abstract

This paper aimed to clarify the association of mid-adolescent dietary practices and experiences with adult eating behavior and attitudes using individual data from the “Survey of Attitudes toward *Shokuiku* (food and nutrition education), 2019” put forth by the Ministry of Agriculture, Forestry and Fisheries of Japan. We applied conditional mixed-process models to estimate the parameters simultaneously, and used them to predict current eating consciousness, current eating behaviors in a balanced diet, dietary behavior, and attitudes toward preventing or improving lifestyle-related diseases as dependent variables. As a result, compared to those who did not have good dietary practices and experiences in mid-adolescence, participants who had good dietary practices and experiences in the same period displayed greater interest in practicing a healthier diet. These participants frequently consumed a combination of staple foods, main dishes, and side dishes, and were more concerned about preventing or improving lifestyle-related diseases. In conclusion, mid-adolescent dietary practices and experiences had a lasting influence on adult eating behaviors and attitudes in Japanese participants.

## 1. Introduction

In many countries, the prevalence of overweight and obese individuals has increased or maintained higher numbers, irrespective of the economic state of the countries [1,2,3,4]. This is the case in Japan as well. According to the National Health and Nutrition Survey in Japan [5,6], the rates of obese female adults aged 20 years or above, whose body mass index is more than 25 kg/m^2^, remained stable between a narrow range of approximately 19–23% during 1980–2017. However, those of their male counterparts drastically increased from 17.8% in 1980 to 22.3% in 1990, 26.8% in 2000, 30.4% in 2010, and 33.0% in 2019 [5,6]. Overweight and obesity are risk factors for non-communicable diseases such as cardiovascular diseases, diabetes mellitus, and cancer [7,8]. In such situations, concerns have grown over the prevalence of obesity or metabolic syndrome in Japan [9,10,11,12]. Since April 2008, all health insurers, such as the National Health Insurance and Employee’s Health Insurance, have been required to provide insured persons and their dependents aged 40 years and above with health checkups, and guidance to prevent obesity or metabolic syndrome among the public in Japan.

Despite such endeavors, the upward trends in the prevalence of obesity among male adults and other nutrition-related issues for both male and female adults remain unsolved. The nationwide health and nutrition surveys showed that, for adults, vegetable intake was approximately 20% lower than the government-recommended amount (350 g/day) [6]. Additionally, more than 50% of adult respondents did not eat a nutritionally balanced combination of staple, main, and side dishes every day [13]. Furthermore, younger adults were less likely to eat nutritionally balanced meals with staple, main, and side dishes [13]. Moreover, female adults in their 20s and 30s tend to consume less protein, calcium, dietary fiber, and potassium than those aged 60 years or older [14]. The prevalence of malnutrition among elderly adults has also been reported [15]. Therefore, regardless of age and sex, improving adult eating behavior is an urgent public health issue in Japan.

As observed in other countries, previous empirical cross-sectional studies in Japan have pointed out many factors affecting adult eating behaviors. These include differences in dietary awareness, and food choices, sex [16,17,18], age [17,18,19], family structure [16,18,19], socioeconomic status [18,19,20,21,22,23], eating with family [18], mental stress [24], mindfulness [25], food/eating literacy [26], accessibility to food outlets [27,28], and mobility restriction due to the coronavirus 2019 (COVID-19) pandemic [29,30].

However, to the best of our knowledge, whether adolescent or childhood feeding and eating practices have a lasting influence on adult eating behaviors and attitudes in Japan has not been investigated. There are only a few exceptional studies using two-stage random sampling survey data [19,31,32,33] and simple random survey data [34] that discussed the topic. Research has shown that Japanese female adults aged 20–59 years with more cooking experience in childhood tend to engage in more favorable eating behaviors and attitudes during adulthood [19]. It has been reported that enjoyable eating, mealtime atmosphere, and mealtime food-related conversation at home during childhood promote healthy eating behaviors and subjective diet-related quality of life in adulthood among Japanese adults aged 20 years or older [31,32,33]. It has also been pointed out that mothers who usually enjoyed a better mealtime atmosphere at home in their primary school period are more likely to have better food consciousness during adulthood [34]. All these findings suggest that childhood food/eating-related environments and experiences would affect eating behavior and attitudes in adulthood.

However, previous studies [19,31,33] measured the extent of childhood eating habits and cooking experiences using only one simple retrospective question with a Likert-type scale. To measure dietary practices and experience (i.e., eating atmosphere, participating in food preparation, receiving guidance about food) more comprehensively and accurately, responses to several multifaceted questions must be used for analysis. In addition, although multiple dependent variables of eating behavior and attitudes among adults have been used in previous studies [19,31], they were analyzed separately, and methods that consider the relationship between these factors were not applied. Therefore, the purpose of this study was to measure the extent to which adolescent dietary practices and experiences are favorable using a wide variety of questions and their answers, and to clarify whether dietary practices and experiences during adolescence are related to eating behavior and consciousness in adulthood in Japan, considering the association between the explained variables. This paper focuses on mid-adolescence or the junior high school period (13–15 years) among adolescents, defined by the WHO as those 10–19 years of age [35], because dietary behavior changes largely during this period.

## 2. Materials and Methods

### 2.1. Data

#### 2.1.1. Data Source and Subjects

The raw data used in the present study were obtained from the “Survey of Attitudes toward *Shokuiku* (food and nutrition education), 2019,” which was conducted by the Ministry of Agriculture, Forestry and Fisheries of Japan in October 2019. Every year, in line with the *Shokuiku* Basic Act, the government must submit a report on measures taken by the government to promote *Shokuiku*. The regulatory agency responsible for this program is the Ministry of Agriculture, Forestry and Fisheries. The ministry conducts surveys of consumers for this purpose. A face-to-face survey was conducted using two-stage random sampling. Altogether, 3000 participants aged 20 years or older who lived in 210 primary sample units of the Japanese population were selected. In total, 1721 respondents (response rate: 57.4%) agreed to be interviewed individually by trained interviewers who asked questions following the completion of the questionnaire. Of the 1721 respondents, individual data from 1569 (679 males and 890 females) participants, for whom all data for the variables used in the analysis were obtained, were used in the discussion. The questionnaire included questions on eating behavior and nutrition education. It also included demographic information such as age, gender, marital status, number of household members, occupations, and regions. The data are not longitudinal and there are limitations in analyzing the causality. However, the data provide credible answers for quantitative analysis as the survey was planned and credited by the Ministry of Agriculture, Forestry and Fisheries of Japan, and professional staff conducted face-to-face interviews with the subjects.

#### 2.1.2. Eating Behaviors and Consciousness in Adulthood

Current eating consciousness was assessed based on the participants’ self-reports by asking the following question: “Are you committed to practicing a healthy diet on a regular basis?” The responses were recorded on a 4-point Likert scale, ranging from “always committed” (1) to “not committed at all” (4). For analysis purposes, the responses were scored in the inverse direction (i.e., always committed = 4, not committed at all = 1).

After the interviewers presented a brief description of staple foods (rice, bread, noodles, etc.), main dishes (made with meat, fish, eggs, soy products, etc.), and side dishes (made with vegetables, mushrooms, potatoes, seaweed, etc.), participants were asked the following question about consuming a balanced diet: “How many days per week do you eat more than two meals with staple food, main dishes, and side dishes?” The responses were recorded on a scale that included “almost every day,” “4 or 5 days per week,” “2 or 3 days per week,” and “a few days per week.” For analysis, the responses were scored in the inverse direction (i.e., almost every day: 6–7; 4 or 5 days per week: 4–5; 2 or 3 days per week: 2–3; a few days per week: 0–1).

Dietary behaviors and attitudes related to lifestyle-related diseases in adulthood were assessed based on participants’ self-reports by asking the following two questions: “To prevent or improve lifestyle-related diseases, to what extent do you pay attention to ….?” and “To prevent or improve lifestyle-related diseases, to what extent do you practice ….?”. Each question was composed of six items (“Regulating energy (calories)”, “Avoiding too much salt (reduce salt intake)”, “Regulating the quantity and quality of fat”, “Avoiding too many sweets (sugar)”, “Eating plenty of vegetables”, and “Eating fruit”). For dietary attitudes toward preventing or improving lifestyle-related diseases, the responses were recorded on a 4-point Likert-type scale ranging from “very careful” (1) to “not careful at all” (4). For analysis purposes, the responses were scored in the inverse direction (i.e., very careful = 4, not careful = 1). Similarly, for dietary behaviors to prevent or improve lifestyle-related diseases, the responses were scored in the inverse direction (i.e., always practicing = 4, not practicing at all = 1).

Since multiple questions were set for dietary behavior and attitudes related to lifestyle-related diseases, the first principal component score obtained by applying categorical principal component analysis (Supplementary Tables S1 and S2) was used for analysis.

#### 2.1.3. Adolescent Dietary Practices

Dietary practices and experiences during adolescence were assessed by asking participants to respond to the following statement: “About your eating habits/experiences during your junior high school years, choose one option that applies to you.” The question was composed of nine items (“At home, you ate all three meals a day at regular times,” “At home, you ate together with your family,” “You did grocery shopping with your family,” “At home, you helped prepare meals and clean up afterward,” “At home, you greeted each other with *Itadakimasu* (Let’s eat) and *Gochisosama* (Thank you for the meal),” “At home, seasonal foods and dishes were prepared according to the season,” “You experienced activities related to food production, such as rice planting and vegetable harvesting at home, at school, and in the community,” and “At school, teachers talked about food and gave you guidance”). 

Since multiple questions were set for adolescent dietary practices and experiences, the first principal component score obtained by applying categorical principal component analysis (Supplementary Table S3) was used for analysis.

#### 2.1.4. Other Covariates

We considered the following covariates: age, sex, subjective economic status, subjective health status, the dummy for living with family and eating together, the interaction term between adolescent eating habits (the first principal component score) and the dummy for living with family and eating together, and the area of residence (large city: 23 wards of Tokyo; large city: an ordinance-designated city; mid-sized city with more than 100,000 people; small city with fewer than 100,000 people; towns or villages) were used for the analysis.

### 2.2. Statistical Analysis

For dietary behavior and attitudes toward preventing or improving lifestyle-related diseases and adolescent eating habits, categorical principal component analysis (CATPCA) was appropriate for data reduction. This was chosen as all these variables are ordinal and we were concerned with identifying the underlying components of a set of items while maximizing the amount of variance accounted for by the principal components.

Given that responses regarding attention to healthy dietary practices are ordinal in nature, an ordered probit regression was used to examine the effects of adolescent dietary practices on attention to healthy dietary practices in adulthood. Since the responses to the question on the frequency of eating nutritionally balanced food were interval in nature, interval regression was applied. Ordinary least squares (OLS) was used to examine the effects of dietary behavior and attitudes toward preventing or improving lifestyle-related diseases in adulthood, which were calculated using CATPCA.

When estimating the parameters of the four equations, correlations may exist between the error terms of each equation, which do not affect the unbiasedness but result in a decrease in the efficiency of the estimators [36]. Therefore, we applied Roodman’s [37] *cmp (conditional mixed process)* command of Stata version 17.0 (StataCorp LLC, College Station, TX, USA) for data analysis, with a significance level of *p* < 0.05.

The conditional mixed-process models presented by Roodman [37] to estimate the parameters simultaneously were used to predict current eating consciousness, current eating behaviors in a balanced diet, dietary behavior, and attitudes toward preventing or improving lifestyle-related diseases as dependent variables. This was carried out by using adolescent eating habits as independent variables, which were adjusted for age, gender, subjective economic status, subjective health status, the dummy for living with family and eating together, the interaction term between adolescent eating habits and the dummy for living with family and eating together, and area of residence.

## 3. Results

### 3.1. Subjects’ Characteristics

The frequencies and means of the independent variables are listed in Table 1. Of the 1569 respondents, 679 were male (43.3%) and 890 were female (56.7%). In terms of the age structure, the 20–24 years old cohort accounted for 4.0%, 25–29 years for 4.6%, 30–34 years for 5.5%, 35–39 years for 7.3%, 40–44 years for 7.6%, 45–49 years for 10.0%, 50–54 years for 9.1%, 55–59 years for 7.1%, 60–64 years for 8.7%, 65–69 years for 10.1%, and >70 years old for 26.1% (only the data for the age cohort were provided by the Ministry of Agriculture, Forestry and Fisheries because of the need to protect the privacy of the research subjects). In terms of cohabitation and eating status, 20.1% of the respondents lived with family but ate alone, 67.9% lived and ate with family, and 12.0% lived and ate alone. As for the area of residence, the large city (23 wards of Tokyo) accounted for 5.9%, large cities (ordinance-designated cities) for 22.1%, mid-sized cities (>100,000 people) for 40.9%, small cities (≤100,000 people) for 21.8%, and towns or villages for 9.3%.

The 5-point Likert scales of subjective economic status and subjective health status were 0.4 ± 1.0 and 0.7 ± 0.9, respectively.

### 3.2. Regression Results

The estimation results are presented in Table 2. The null hypothesis that “the coefficients of all explanatory variables except the constant term are equal to zero” was rejected at the 1% level (the test statistic following a chi-square distribution with 88 degrees of freedom was 653.54). In addition, the signs of the estimated coefficients are generally consistent with those reported in previous studies, suggesting that the estimation results are relatively good. Regarding the correlations among the error terms across the four estimated equations, all six correlation coefficients were significant at the 1% level. Therefore, it seems more appropriate and accurate to use the method presented by Roodman [37] to estimate the parameters simultaneously rather than solving the four equations separately.

Table 2 clearly shows that, given the other conditions being the same for the dummies for the >45 years old age groups, the coefficients were significantly positive compared to their 20–24-year-old counterparts for attention to healthy dietary practices, frequency of eating nutritionally balanced food, attention to preventing lifestyle diseases, and dietary practices for preventing lifestyle diseases. Moreover, compared to the male sex, the female sex was found to be positively significant at the 5% credential level for all dependent variables related to dietary awareness and practices. With regard to subjective health status, the coefficients were significantly positive for attention to healthy dietary practices, attention to preventing lifestyle diseases, and dietary practices for preventing lifestyle diseases.

Regarding the adolescent dietary practices, given other conditions being the same, the coefficients of dietary practices in junior high school were significantly positive for attention to healthy dietary practices, frequency of eating nutritionally balanced food, and attention to preventing lifestyle diseases. As for the dummies for living with family and eating together, given other conditions being the same, compared to people who were living together with family but eating alone, those who were living and eating together with family were found to have better eating behavior and consciousness for all dependent variables. On the other hand, the participants who were living and eating alone displayed a lower frequency of eating nutritionally balanced food. In addition, the interaction variables between dietary practices in junior high school and the dummy for living with family and eating together were significant with positive coefficients for attention to preventing lifestyle diseases and dietary practices for preventing lifestyle diseases. 

## 4. Discussion

The analysis using the CMP model revealed that those with high dietary awareness and good dietary practices in adulthood generally fulfilled the following conditions: (1) middle-aged, (2) female, (3) financially affluent, (4) good health, (5) good dietary practices in mid-adolescence (junior high school period), and (6) currently cohabiting and eating with family. The conclusions are summed up in Table 3.

While expanding the details on the fifth condition, the present study postulated that a person had good dietary practices in mid-adolescence if he/she had meals regularly, ate meals with their family, shopped for groceries with their family, helped their family prepare meals and clean up after meals at home, greeted before and after meals at home, had seasonal foods and dishes at home, enjoyed mealtime at home, experienced farm practices, and had a class regarding food and nutrition at school. Compared to those who did not have good dietary practices and experiences in junior high school, the participants who had good dietary practices and experiences in the same period showed greater interest in practicing a healthier diet. These participants frequently consumed a combination of staple foods, main dishes, and side dishes, and were more concerned about preventing or improving lifestyle-related diseases.

In general, the results are consistent with the findings of the limited number of previous studies that showed relevance in the relationship between adolescent and adult dietary practices in Japan and other countries. In the USA, it has been pointed out that the more frequently adults eat with their families during adolescence, the higher the quality and frequency of their diets during their youth [38]. It has also been shown that adults who ate meals regularly in childhood tend to eat meals regularly in their adulthood (in the USA) [39] and do not skip breakfast (in Japan) [33]. Several Japanese studies have shown that enjoyable eating and a good mealtime atmosphere at home during childhood promoted healthy eating behaviors, subjective diet-related quality of life, and food consciousness in adulthood [31,32,34]. It has been pointed out that caregivers talking about nutrition during childhood affected food choices and nutritional considerations among college students in the USA [39]. Research has shown that Japanese adults who receive nutrition education at junior high school are more likely to be interested in nutrition education in their adulthood [32].

Significant changes in dietary behaviors in adolescence, such as an increased prevalence of skipping breakfast, increased consumption of soft drinks, and decreased consumption of fruit and vegetables, have been widely reported [40,41,42]. These have been attributed to increased autonomy in food choice and the relative importance of relationships with friends compared to that with family [43]. In addition, dietary intake in adolescence is a significant but weak predictor of intake in adulthood [44]. However, some studies have found a strong relationship between dietary intake patterns during adolescence and adulthood [42,45,46]. Longitudinal studies in Norway [42] and Portugal [45] have shown that dietary intake patterns in mid-adolescence (ages 13-14) and young adulthood (21 years) are highly similar. Another longitudinal study in Finland [46] showed that diet in childhood (ages 3-18) affected the diet of the participants in adulthood (21 years later). Unlike these earlier studies, the present paper did not compare the intake patterns of specific food groups such as vegetables, fruits, meat, seafood, soft drinks, and fast food between adolescence and adulthood. It is clear that adults with favorable eating habits and food experiences during mid-adolescence are more likely to engage in more favorable dietary practices and have higher food consciousness in Japan.

There are several possible reasons for the long-term effects of adolescent dietary practices on adult dietary habits and food consciousness. The effect of the socioeconomic status of a family on dietary practices cannot be ignored. In general, it has been shown that children from families with a lower socioeconomic status tend to have aberrant eating behaviors, such as skipping breakfast, picky eating, and consumption of unhealthy foods and beverages [47,48]. A study on British participants showed that diet quality in older men is influenced by socioeconomic factors in childhood and adulthood [49]. Recent studies have indicated the existence of intergenerational transmission of socioeconomic status [50,51]. Considering these points together, we cannot deny the possibility that subjects who grew up in economically deprived households tend to have unfavorable dietary practices during their adolescence and are unable to overcome economic deprivation in adulthood due to socioeconomic deprivation [50,51]. This leads to unfavorable dietary practices in adulthood. Although we could not examine the details from the cross-sectional and retrospective survey data, some of the components of adolescent dietary practices used in the present study were less likely to be directly related to socioeconomic status. Therefore, although the effect of intergenerational transmission of socioeconomic status cannot be denied, it is also possible that the formation of adolescent dietary practices directly influences dietary practices and food awareness in adulthood, even after removing the effects of intergenerational transmission of socioeconomic status. 

Regardless of the reasons for the influence of adolescent dietary practices and experiences on adult dietary habits, our analyses show that improving adolescent dietary practices may be effective in improving dietary habits and possibly preventing adult lifestyle-related diseases in the future. This also suggests that regular meals, eating or cooking meals with family, enjoyable mealtimes, farm experience, and nutrition education at school could be some of the key practices to acquire favorable dietary habits in adulthood. However, in today’s Japanese society, it is difficult to eat or cook together because of lifestyle changes such as the increase in dual-earner households [52], long working hours [53], and the number of children attending cram schools [54]. It has been suggested that having a meal with the family can lead to conversation with the family members, improve the dining atmosphere [55], and help to develop healthier dietary and eating patterns [56]. Therefore, although the Amsterdam Growth and Health Longitudinal Study suggested that dietary intake is changeable [57], it is important to have opportunities to share the dining table with the family during adolescence. In addition, it may be important to provide adolescents with opportunities to think about how eating habits should be at home, not only from the perspective of nutritional intake but also by specifically addressing overall eating habits, such as menu planning, eating together, helping, and conversation.

This study has several limitations. First, although this study used the combined index of mid-adolescent dietary practices for analysis, all information during the study period was based on answers to retrospective questions. Therefore, it is possible that accurate response results may not have been obtained because of recall errors by the participants. Second, information on the economic conditions of the participants’ families, their parents’ educational attainment and occupation, and family structure during adolescence was not available; therefore, we were unable to fully examine the complicated mechanisms whereby adolescent dietary practices affect those in adulthood from the retrospective, cross-sectional survey data. For a more detailed analysis, it will be necessary to consider whether adolescent dietary practices are affected by the socioeconomic status of the families at that time. Dietary practices during adulthood were determined by the current socioeconomic status and generational transmission of socioeconomic status. To overcome these limitations, a panel data analysis using longitudinal data is necessary. Third, in the present paper, adults across a wide age group (20 years and older) were analyzed. Therefore, the environment associated with food and diet, common sense regarding dietary intake, available food at outlets, and family composition differed greatly depending on the adolescents’ time they spent. Finally, the research response rate was somewhat low, and whether there were any specific groups that did not respond to the questionnaire is unclear. Therefore, caution should be exercised when interpreting the results of this study. Further research based on the latest dataset with detailed long-term information on subjects is required to examine the long-term influence of adolescent dietary practices on eating behaviors and attitudes in adulthood.

## 5. Conclusions

We evaluated the effects of mid-adolescent dietary practices on eating behaviors and attitudes in adulthood. The results highlight that the participants who had good dietary practices and experiences in mid-adolescence showed greater interest in practicing a healthier diet than those who did not. Therefore, parents and schools should be provided with information and guidance on reasons and ways to actively engage in the nutrition education of adolescents and their dietary life so that the dietary practices and experiences of children in mid-adolescence, as well as their eating behaviors and attitudes in adulthood, is improved.

## Figures and Tables

**Table 1:** Subject characteristics.

Characteristics	Total = 1569	
Age cohort		
20–24 years	63	(4.0)
25–29 years	72	(4.6)
30–34 years	86	(5.5)
35–39 years	114	(7.3)
40–44 years	119	(7.6)
45–49 years	157	(10.0)
50–54 years	142	(9.1)
55–59 years	112	(7.1)
60–64 years	137	(8.7)
65–69 years	158	(10.1)
70+ years	409	(26.1)
Sex		
Male	679	(43.3)
Female	890	(56.7)
Subjective economic status	0.4	SD = 1.0
Subjective health status	0.7	SD = 0.9
Dietary habits in junior high school (principal component scores)	0.0	SD = 1.8
Dummy for living with family and eating together		
Living together but eating alone	315	(20.1)
Living together and eating together	1066	(67.9)
Living alone and eating alone	188	(12.0)
Area of residence		
Large city: 23 wards of Tokyo	93	(5.9)
Large city: ordinance-designated city	347	(22.1)
Mid-sized city: >100,000 people	641	(40.9)
Small city: ≤100,000 people	342	(21.8)
Towns or villages	146	(9.3)

**Table 2:** Estimation results.

Dependent/Independent Variables	Attention to Healthy Dietary Practices (Ordered Probit)			Frequency of Eating Nutritionally Balanced Food (Interval Regression)			Attention to Preventing Lifestyle Diseases (Principal Component Scores)			Dietary Practices for Preventing Lifestyle Diseases (Principal Component Scores)		
	Coefficient	Standard Error		Coefficient	Standard Error		Coefficient	Standard Error		Coefficient	Standard Error	
Age cohort (reference: 20-24 years)												
25–29 years	0.480	0.192	*	0.205	0.311		0.637	0.285	*	0.526	0.291	
30–34 years	0.615	0.184	**	0.166	0.299		0.478	0.274		0.330	0.280	
35–39 years	0.675	0.175	**	0.321	0.283		1.003	0.259	**	0.747	0.265	**
40–44 years	0.868	0.174	**	0.408	0.281		1.203	0.257	**	1.040	0.263	**
45–49 years	0.738	0.167	**	0.623	0.269	*	1.279	0.247	**	1.103	0.252	**
50–54 years	0.995	0.170	**	0.841	0.274	**	1.707	0.251	**	1.558	0.256	**
55–59 years	0.947	0.177	**	0.940	0.285	**	1.759	0.260	**	1.630	0.266	**
60–64 years	0.822	0.170	**	0.979	0.275	**	1.495	0.252	**	1.475	0.258	**
65–69 years	1.054	0.169	**	1.099	0.272	**	1.916	0.249	**	1.858	0.254	**
>70 years	1.345	0.155	**	1.547	0.248	**	1.980	0.227	**	2.096	0.232	**
Sex (reference: male)												
Female	0.332	0.060	**	0.243	0.096	*	0.414	0.088	**	0.324	0.090	**
Subjective economic status	0.098	0.029	**	0.160	0.046	**	0.180	0.042	**	0.189	0.043	**
Subjective health status	0.118	0.033	**	0.103	0.054		0.123	0.049	*	0.167	0.050	**
Dietary practices in junior high school (principal component scores)	0.111	0.035	**	0.160	0.057	**	0.116	0.052	*	0.087	0.053	
Dummies for living with family and eating together (reference: living together but eating alone)												
Living together and eating together	0.159	0.075	*	0.427	0.121	**	0.270	0.111	*	0.244	0.113	*
Living alone and eating alone	0.147	0.107		-0.366	0.172	*	0.139	0.157		0.098	0.161	
Dietary practices in junior high school × dummies for living with family and eating together												
Dietary practices in junior high school × living together and eating together	0.018	0.040		0.005	0.065		0.120	0.059	*	0.158	0.060	**
Dietary practices in junior high school × living alone and eating alone	−0.040	0.057		0.007	0.092		0.067	0.084		0.124	0.086	
Area of residence (reference: towns or villages)												
Large city: 23 wards of Tokyo	0.015	0.149		0.229	0.241		0.324	0.220		0.446	0.225	*
Large city: ordinance-designated city	0.205	0.110		0.255	0.178		0.072	0.163		0.269	0.166	
Mid-sized city: >100,000 people	−0.008	0.103		−0.018	0.166		0.027	0.152		0.166	0.155	
Small city: ≤100,000 people	0.059	0.111		0.063	0.178		0.108	0.163		0.278	0.167	
Cutoff 1	−0.870	0.184	**									
Cutoff 2	0.657	0.179	**									
Cutoff 3	2.178	0.184	**									
Constant				3.541	0.287	**	−2.129	0.263	**	−2.174	0.269	**
Sigma				1.767	0.032	**	1.638	0.029	**	1.675	0.030	**

Note: ** and * denote significance at the 1% and 5% level, respectively.

**Table 3:** Estimation results *.

Factors	Attention to Healthy Dietary Practices	Frequency of Eating Nutritionally Balanced Food	Attention to Preventing Lifestyle Diseases	Dietary Practices for Preventing Lifestyle Diseases
Age cohort	○	△	△	△
Sex	○	○	○	○
Subjective economic status	○	○	○	○
Subjective health status	○	╳	○	○
Dietary practices in junior high school	○	○	○	╳
Dummies for living with family and eating together	△	○	△	△
Dietary practices in junior high school × dummies for living with family and eating together	╳	╳	△	△
Area of residence	╳	╳	╳	△

*: ○, significant; △, partly significant; ╳, insignificant.

## Data Availability

Raw data are publicly available upon request from the Japan Social Science Data Archive, Center for Social Research and Data Archives, Institute of Social Science, University of Tokyo, Japan.

## Supplementary Materials

The following are available online at https://www.mdpi.com/article/10.3390/nu15010225/s1, Table S1: Principal component analysis on the degree of attention to preventing or improving lifestyle-related diseases; Table S2: Principal component analysis on the degree of practicing to prevent or improve lifestyle-related diseases; Table S3: Principal component analysis of dietary practices in junior high school.
