# Supplementary material for: Effect of Mid-Adolescent Dietary Practices on Eating Behaviors and Attitudes in Adulthood
Source: Nutrients. 2023 Jan 1;15(1):225. doi: 10.3390/nu15010225 (PMC9823809; doi:10.3390/nu15010225)
Supplement: Supplementary file 1 [file nutrients-15-00225-s001.zip › nutrients-2058500-supplementary.pdf]

Supplementary Materials

Effect of Mid-Adolescent Dietary Practices on Eating Behaviors and Attitudes in Adulthood

Miao Wu, Lin Wu and Akira Ishida *

Results of Principal Component Analysis

Principal component analysis was applied to the data of the responses to the six items on ‘‘To prevent or improve lifestyle-related diseases, to what extent do you pay attention to?’’. In the first principal component, the factor loadings of the six items all showed the same level of positive values, and the first principal component score obtained was set as "the extent to care about preventing and improving lifestyle-related diseases". For the second principal component, the factor loadings were highly positive for two items related to vegetables and fruit intake, and negative for four items related to calories, salt, fat, and sugar, which should be avoided in daily life. Therefore, "the extent to consume vegetables and fruit" was set. However, considering that the contribution rate of the first principal component (61.9%) was much higher than that of the second principal component (13.9%), only the first principal component score, "the extent to care about preventing and improving lifestyle-related diseases" was used in the analysis (Table S1). Similarly, the contribution rate of the first principal component (65.7%) was significantly higher than that of the second principal component (12.6%) for "practice in prevention and improvement of lifestyle-related diseases", and only the first principal component score was selected to prevent or improve lifestyle-related diseases (Table S2).

Table S1. Principal component analysis on the degree of attention to preventing or improving lifestyle-related diseases

	Principal Component 1	Principal Component 2
Eigenvector		
Regulating energy (calories)	0.417	-0.324
Avoiding too much salt (reducing salt intake)	0.439	-0.136
Adjusting the quantity and quality of fats (fat)	0.450	-0.223
Avoiding too many sweets (sugar)	0.405	-0.330
Eating plenty of vegetables	0.382	0.504
Eating fruit	0.347	0.681
Eigenvalue	3.716	0.831
Contribution ratio [1]	0.619	0.139

[1] Contribution ratio = Eigenvalue / number of items.

Table S2. Principal component analysis on the degree of practicing to prevent or improve lifestyle-related diseases

	Principal Component 1	Principal Component 2
Eigenvector		
Regulating energy (calories)	0.410	-0.349
Avoiding too much salt (reducing salt intake)	0.440	-0.139
Adjusting the quantity and quality of fats (fat)	0.448	-0.230
Avoiding too many sweets (sugar)	0.405	-0.317
Eating plenty of vegetables	0.379	0.529
Eating fruits	0.361	0.653
Eigenvalue	3.941	0.756
Contribution ratio	0.657	0.126

Principal component analysis was applied to the data of nine items related to "dietary practices in junior high school". The factor loadings of the nine items in the first principal component were all positive to the same extent. Considering that the contribution rate of the first principal component (41.3%) was significantly higher than that of the second principal component (14.4%), approximately 40% of the total information was condensed into the first principal component. Only the first principal component score was used in the analysis (Table S3).

Table S3. Principal component analysis of dietary practices in junior high school

	Principal Component 1	Principal Component 2
Eigenvector		
At home, all three meals a day were eaten at a fixed time	0.335	-0.451
Had meals together with family at home	0.380	-0.355
Grocery shopped with family at home	0.293	0.413
Helped prepare and clean up after meals at home	0.309	0.363
At home, said "*Itadakimasu*" and "*Gochisosama*"	0.324	-0.007
At home, seasonal foods and dishes were prepared	0.380	-0.247
At home, mealtimes were pleasant and comfortable	0.397	-0.140
Experienced activities related to food production at home, at school, and in the community	0.275	0.252
At school, received guidance about food from teachers	0.283	0.474
Eigenvalue	3.713	1.295
Contribution ratio	0.413	0.144
